# A retrospective study of adjuvant proton radiotherapy for breast cancer after lumpectomy: a comparison of conventional-dose and hypofractionated dose

**DOI:** 10.1186/s13014-023-02213-8

**Published:** 2023-03-23

**Authors:** ZhengShan Hong, ZhaoZhi Yang, Xin Mei, Ping Li, Cihang Bao, Zheng Wang, Xin Cai, Xue Ming, WeiWei Wang, XiaoMao Guo, XiaoLi Yu, Qing Zhang

**Affiliations:** 1 Department of Radiation Oncology, Shanghai Proton and Heavy Ion Center, 4365 Kangxin Road, Pudong, Shanghai, 201321 China; 2 Shanghai Key Laboratory of Radiation Oncology (20dz2261000), Shanghai, China; 3 Shanghai Engineering Research Center of Proton and Heavy Ion Radiation Therapy, Shanghai, China; 4 Department of Radiation Physics, Shanghai Proton and Heavy Ion Center, Shanghai, China; 5 Department of Radiation Oncology, Fudan University Shanghai Cancer Center, Shanghai, China; 6 Department of Oncology, Shanghai Medical College, Fudan University, Shanghai, China

**Keywords:** Proton radiotherapy, Breast cancer, Lumpectomy

## Abstract

**Purpose:**

This study aimed to compare the adverse reactions of conventional-dose and hypofractionated dose of proton therapy for breast cancer.

**Materials and methods:**

Breast cancer patients treated with proton radiotherapy in conventional-dose or hypofractionated dose were studied retrospectively.

**Result:**

From January 2017 to December 2019, our center treated 50 patients following lumpectomy with proton radiotherapy. According to the AJCC 8th Edition standard, there were stage I in 26 patients, stage II in 22 patients, and stage III in 2 patients. A total of 14 patients received intensity-modulated proton therapy at a dose of 50 Gy in 25 fractions, followed by a 10 Gy 4 fractionated boost to the lumpectomy cavity, while 36 received 40.05 Gy in 15 fractions, simultaneous integrated boost (SIB) 48 Gy to the lumpectomy cavity. Median follow-up time for 40.05 Gy group was 35.6 months (15–43 months). Median follow-up time for 50 Gy group was 46.8 months (36–68 months). For acute toxicity, the grade 1 and 2 radiodermatitis in conventional-dose group were 35.7% and 57.1%, respectively. In hypofractionated dose group, the grade 1 and 2 radiodermatitis were 91.7% and 8.3%, respectively. The radiodermatitis is hypofractionneted dose better than conventional-dose significantly. Grade 1 radiation-induced esophagitis in conventional-dose group and hypofractionated dose group were 85.71% and 60%, respectively. For late toxicity, no patients developed radiation-induced pneumonitis and rib fracture in conventional-dose group. Three patients presented grade 1 pneumonitis; one patient presented graded 2 pneumonitides and two patients presented rib fracture in hypofractionated dose group. One presented hypothyroidism in hypofractionated dose group. All patients were satisfied with breast shape. The one- and two-year OS and DFS for conventional-dose group were 100 and 100; 100 and 92.9%, respectively. The one- and two-year OS and DFS for hypofractionated dose group were 100 and 100; 100 and 100%, respectively.

**Conclusion:**

Proton radiation therapy can significantly reduce the normal tissue dose in breast cancer patients' hearts, lungs, and other organs. Hypofractionated proton therapy shortens the treatment course with mild radiation-related adverse effects, and has a better effect on addressing the acute adverse reactions than conventional proton radiotherapy.

## Background

Breast cancer is the most common cancer in women, with about 1.15 million new cases diagnosed annually. Among all therapeutic methods, surgical operation, chemotherapy, and radiation therapy are the three most common in the medical community. After systemic treatment, patients may have a higher chance of survival. According to the Lancet publication in 2018, Global surveillance of trends in cancer survival 2000–14, the five-year survival rate of breast cancer in China has reached around 80% [1]. Aside from breast cancer patients receiving a satisfactory survival following appropriate treatment, another critical criterion, quality of life, is becoming increasingly appealing, prompting many doctors and researchers to focus on improving patients' quality of life. Surgery are focus on develop breast reconstruction technique that can reduce patient surgical trauma. Radiation therapy focus on how to alleviate radiation induced side effects.

Radiation therapy is an important treatment option for breast cancer patients. The chest wall and tumor bed are the most common site of breast cancer recurrence after surgery, accounting for 48–60% of all cases [2, 3]. Adjuvant radiation therapy reduces the local recurrence rate of ten years from 31 to 15.6% in breast cancer after lumpectomy [4] and the locoregional recurrence from 32.1 to 13% in breast cancer after mastectomy [5]. The most common side effect of radiation therapy in breast cancer cases is skin reaction. Radiation also affects the heart and lungs later. In the follow-up of patients with breast cancer subject to radiation therapy, the researchers found that left breast cancer presented further cardiac events, and the incidence rate was related to the mean dose of the heart [6]. According to a New England Journal article, rates of major cardiac events increased linearly with the mean dose to the heart by 7.4% per gray (95% confidence interval, 2.9 to 14.5; *P* < 0.001), with no apparent threshold [7]. Experts contrived to reduce heart and lung exposure doses by improving radiotherapy.

Proton radiation therapy is increasingly used in cancer patients due to the physical benefit of bragg-peak, which exposes a high dose in the tumor region while protecting surrounding organs. A dosimetric study found that proton radiation therapy provided better dose coverage and reduced heart exposure [8].

Usually, radiation therapy uses 2 Gy per day five times a week. In the UK, the Phase III clinical study of START-trial had compared the curative and radiotherapy-related side effects of conventional fractionated adjuvant radiotherapy (50 Gy/25Fx/5W) and hypofractionated adjuvant radiotherapy (39–41.6 Gy/13Fx/5W or 40 Gy/15Fx/3W) after breast cancer surgery. The results demonstrated no statistically significant difference in local recurrence between conventional and hypofractionated radiotherapy. The hypofractionated radiotherapy group presented better skin reaction and cosmetic results than the conventional radiotherapy group [9–12]. According to this study, the ESMO guidelines in 2013 and the NCCN guidelines in 2015 accepted 40–42.5 Gy/15-16FX for whole-breast hypofractionated radiotherapy.

There was no data on hypofractionated proton radiotherapy for the entire breast until now. In 2017, our center began proton therapy for breast cancer. This study compares the side effects and efficacy of conventional proton radiotherapy with hypofractionated proton radiotherapy.

## Materials and methods

From January 2017 to December 2019, 79 breast cancer patients underwent postoperative adjuvant proton radiotherapy at Shanghai Proton and Heavy-ion Center; fifty were post lumpectomy patients. All these 50 patients were included in this study.

### Radiotherapy procedures

**Fig. 1:**
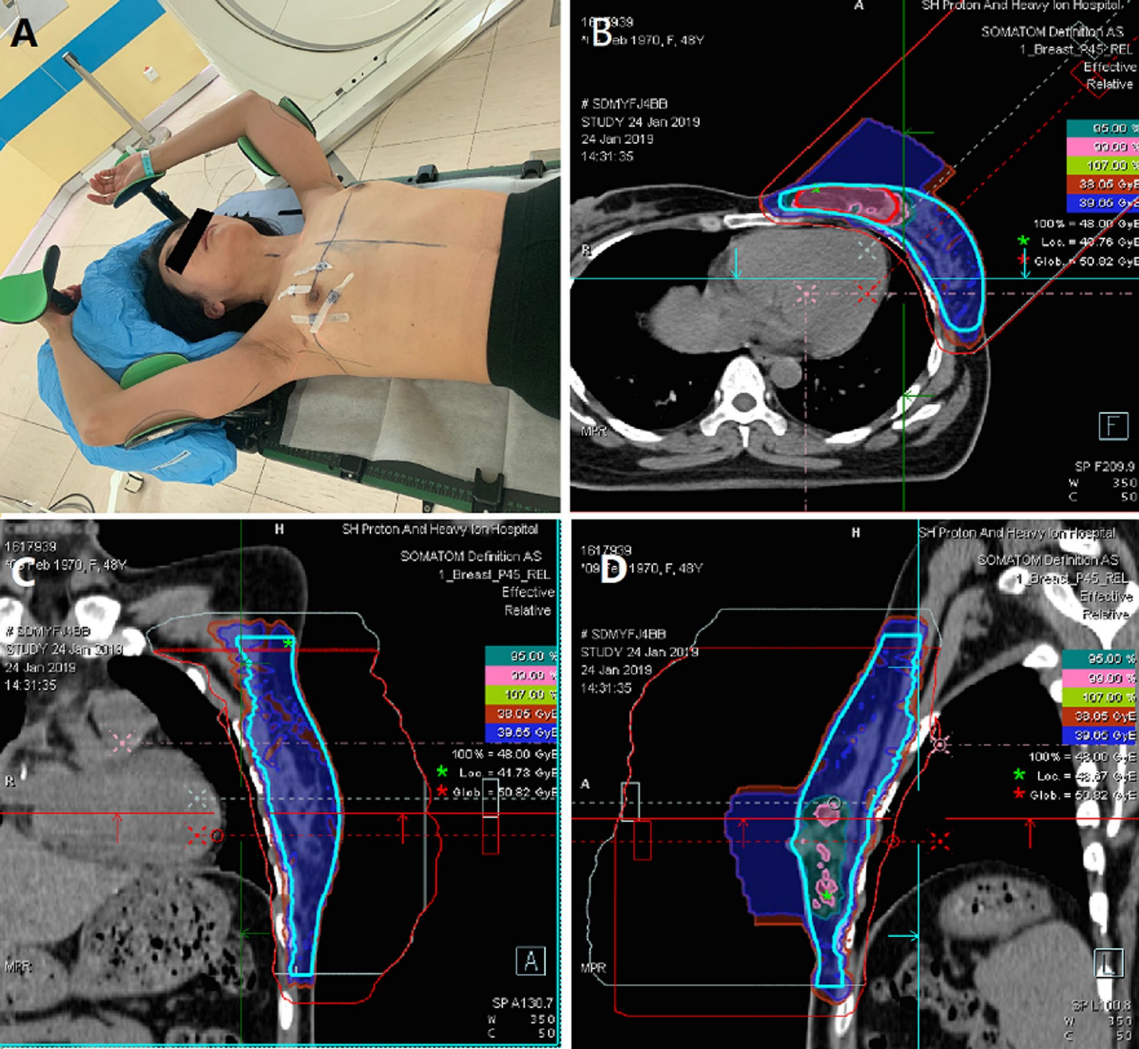
patients immobilization and treatment plan. **A** patient’s immobilization: patients trouble side arm fixed by vacuum pad to improve the repeatability of the position. **B**–**D** The treatment plan with transverse section, coronal section and vertical section

The patients were immobilized supine position with a breast bracket (Fig. 1A). If the supraclavicular region or the neck must be treated, the head must be taken to the contralateral side and then fixed by the head mask. Two types of CT examinations must be used to improve therapy performance. The first is four-dimensional CT, which assesses breast respiratory mobility. The other is plain free-breathing CT, which is used for target countering and treatment planning. (Three surface lead markers were, indeed, used to target the treatment volume prior to CT simulation). The clinical target volumes (CTV) and organs at risk (OAR) were defined according to the breast cancer atlas for radiation therapy planning consensus definitions of the Radiation Therapy Oncology Group (RTOG) (http://www.rtog.org/CoreLab/ContouringAtlases/BreastCancerAtlas.aspx). Planned target volume (PTV) will be expanded from CTV within the body region by adding 5 mm in the lateral direction, 3 mm in the proximal direction, and 3 mm in the distal direction, according to concerning involving the beam uncertainty, set-up uncertainty and range uncertainty of patients. To avoid the high dose irradiating lung tissue, a 2 mm gap between the rear boundary of PTV and lung should be compromised by sacrificing PTV.

The Syngo planning system (Siemens) generated the treatment plan with the intensity-modulated model. The patient treatment used proton pencil scanning beam (Iontrix, Siemens, Germany). The dose prescribed for the whole breast or lymphatic drainage area was 50 Gy in 25 fractions per day of 2 Gy five days per week, followed by a 10 Gy 4 fractionated boost to the lumpectomy cavity or 40.05 Gy in 15 fractions per day of 2.67 Gy five days per week, simultaneous integrated boost (SIB) to 48 Gy per day of 3.2 Gy to the lumpectomy cavity. Each plan was designed to achieve 95% prescription dose coverage of 100% clinical target volumes. During treatment planning, the skin is defined as the first 3 mm of tissue beneath the body's surface and is considered both a target and an organ at risk. To ensure adequate coverage of the dermal lymphatics but to limit the risk of dermatologic toxicity, planning objectives for the skin dose are not less than 90% of the prescription dose. The normal tissue tolerance dose for the 50 Gy group was the ipsilateral mean lung dose ≤ 15 Gy, ≤ 25% of the ipsilateral lung to receive ≤ 20 Gy; ≤ 5% of the heart to receive ≤ 30 Gy, mean heart dose ≤ 1 Gy. For the 40.05 Gy group were ipsilateral mean lung dose ≤ 10 Gy, ≤ 15% of the ipsilateral lung to receive ≤ 20 Gy; ≤ 5% of the heart to receive ≤ 30 Gy, mean heart dose ≤ 1 Gy.

Every patient underwent a verification CT scan before the first treatment and during the first week of therapy to assess the repeatability of the treatment planning and the probability of adaptive re-planning. Before daily treatment, patients take anteroposterior and lateral x-ray photos, after pair the skeleton structure and skin markers, patient started treatment.

### Toxicity

Acute toxicity was defined as adverse reactions occurring within three months after radiotherapy. It is recorded based on CTCAE 4.0. Consideration was given to blood tests, dermatitis, pain, skin infection, and radiation-induced esophagitis. Late toxicity was defined as adverse reactions occurring after three months of radiotherapy. The assessment, according to RTOG, included pneumonitis and rib fracture and so on. The patient describes the breast size, shape, hardness, and arm mobility. The Cosmetic effect were evaluated according to Harvard scale [13]. In each follow-up, the time to resolution of the toxicity was evaluated.

### Follow-up

Follow-up is a clinical examination every three months after proton therapy that includes blood tests, ultrasound mammary gland every three months, breast MR, chest CT, and ECT once a year. The overall survival rate (OS) was calculated from the date of disease diagnosis. Disease-free survival rate (DFS) was calculated from the operation day. The follow-up time was calculated from the date of disease diagnosis.

### Statistics analysis

The Kaplan–Meier method was used to estimate survival and the differences between groups were assessed using the log-rank test. *P* < 0.05 was considered statistically significant for all tests. Analyses were performed using SPSS statistical software (Version 20, IBM SPSS Statistics for macOS).

## Results

### Patients' characteristics

From January 2017 to December 2019, we treated 50 patients after lumpectomy. The patient characteristics are displayed in Table 1. The patient's median age at therapy was 47 years old (29–77 years old). A total of 36 were left breast cancer cases, 12 were right breast cancer cases, and 2 were bilateral breast cancer cases. According to the AJCC 8th Edition standard, there were stage I in 26 patients, stage II in 22 patients, and stage III in 2 patients. A total of 29 patients were estrogen receptor status positive; 26 were progesterone receptor status positive, and eight were her-2 positive. Two patients received neoadjuvant chemotherapy before surgery, 37 received adjuvant chemotherapy before radiotherapy, and 11 did not receive chemotherapy. A total of 14 patients were treated with intensity-modulated proton therapy (IMPT) at a dose of 50 Gy in 25 fractions, followed by a 10 Gy 4 fractionated boost to the lumpectomy cavity, and IMPT treated 36 patients at a dose of 40.05 Gy in 15 fractions per day of 2.67 Gy five days per week, simultaneous integrated boost (SIB) to 48 Gy per day of 3.2 Gy to the lumpectomy cavity. The α/β value for breast tissue is 4.5 Gy, figure out EQD2 for 40.05 Gy/15Fx is 44 Gy. A total of 34 cases treated breast alone; 6 treated axillary lymph node area, 5 included internal mammary lymph node area, and 12 included supraclavicular area. The patient characteristics for 50 Gy group and 40.05 Gy group also displayed in Table 1 respectively. Two sets of data showed 40.05 Gy group distributed more early pathological stage. The reason is, at the beginning we started proton therapy, we treated patients in dose of 40.05 Gy/15Fx who only need treat breast area. Then the literature of hypofractionated photon radiotherapy irradiate lymphatic drainage was gradually increased, therefore later we started irradiate lymphatic drainage area in dose of 40.05 Gy/15Fx. It may influence on compare of the OS and DFS with two groups, but has no effect on the results of the adverse effect of radiotherapy.

**Table 1:** patiens characteristics

Parameter	Total number (50 cases)	50 Gy group (14 cases)	40.05 Gy group (36 cases) (36 cases)	*p*-value
Age, median (range)	29–77(47)	29–65(47)	32–77(47)	0.754
*Area of treatment*				–
Breast only	34	4	30	
Supreclavicular area	12	7	5	
Axillary area	6	4	2	
Internal mammary LN	5	5	0	
*Laterality*				0.403
Left	36	11	25	
Right	12	3	9	
Bilateral	2	0	2	
*Histology*				0.065
Invasive ductal carcinoma	38	13	25	
Invasive lobular carcinoma	4	1	3	
Mixed	4	0	4	
Carcinoma in situ	2	0	2	
Other	2	0	2	
*Estrogen receptor status*				–
Positive	29	6	23	
Negative	21	8	13	
Progesterone receptor status				
Positive	26	5	21	
Negative	24	9	15	
Her2/neu status				
Positive	8	2	6	
Negative	42	12	30	
*Histologic grade*				0.065
1	7	1	6	
2	20	5	15	
3	21	8	13	
Unknown	2	0	2	
*Tumor stage*				–
T1	38	10	28	
T2	11	3	8	
T3	1	1	0	
T4	0	0	0	
*Nodal stage*				–
N0	35	6	29	
N1	14	7	7	
N2	1	1	0	
N3	0	0	0	
*Pathological stage* Stage				0.042
I	26	5	21	
II	22	7	15	
III	2	2	0	
*Chemotherapy timing*				0.119
Adjuvant	37	12	25	
Neoadjuvant	2	1	1	
None(endocrinotherapy)	11	1	10	
*Radiation dose*				–
50 Gy	14	–	–	
40.05 Gy	36	–	–	

### Critical organ dose and target coverage

The dose of OARs and target coverage are illustrated in Table 2. The V95 and V99 for CTV for 50 Gy and 40.05 Gy groups were 99.72 ± 0.28%, 94.13 ± 4.03% and 99.86 ± 0.13%, 94.37 ± 4.66% respectively.

**Table 2:** Target coverage and critical organ dose

	40.05 Gy group	50 Gy group
*CTV(clinical target volume)*		
V95	99.86 ± 0.13%	99.72 ± 0.28%
V99	94.37 ± 4.66%	94.13 ± 4.03%
*Ipsilateral lung*		
V5	23.40 ± 9.73%	30.72 ± 9.95%
V10	17.08 ± 7.10%	23.38 ± 8.49%
V20	8.86 ± 4.31%	13.41 ± 5.91%
V30	3.43 ± 2.36%	6.63 ± 3.74%
V40	0.31 ± 0.55%	2.53 ± 1.97%
V50	–	0.28 ± 0.49%
Dmean	4.53 ± 1.84 Gy	6.61 ± 2.52 Gy
*Contralateral lung*		
V5	0.45 ± 0.79%	2.16 ± 2.17%
V10	0.17 ± 0。01%	0.92 ± 1.11%
V20	0.03 ± 0.07%	0.19 ± 0.31%
V30	0.01 ± 0.00%	0.04 ± 0.09%
V40	–	0.02 ± 0.03%
V50	–	–
Dmean	0.11 ± 0.13 Gy	0.39 ± 0.33 Gy
*Heart (left breast cancer)*		
D2%	9.09 ± 6.19 Gy	15.40 ± 9.90 Gy
V5	3.25 ± 1.98%	4.74 ± 2.87%
V10	1.87 ± 1.29%	3.09 ± 2.20%
V20	0.71 ± 0.66%	1.63 ± 0.85%
V30	0.20 ± 0.24%	0.85 ± 0.31%
V40	0.02 ± 0.04%	0.31 ± 0.31%
V45	–	0.13 ± 0.03%
V50	–	0.03 ± 0.06%
Dmean	0.58 ± 0.33 Gy	0.98 ± 0.63 Gy
*LAD (left anterior descending artery)*		
D2%	17.33 ± 10.98 Gy	28.59 ± 11.05 Gy
Dmean	4.27 ± 3.27 Gy	4.86 ± 2.42 Gy

In left breast cancer, the mean doses for the heart, V5 Gy, and V20 Gy for 50 Gy and 40.05 Gy groups were 0.98 ± 0.63 Gy, 4.74 ± 2.87%, 1.63% ± 0.85% and 0.58 ± 0.33 Gy, 3.25 ± 1.98%, 0.71 ± 0.66% Gy respectively.

In left breast cancer, the mean doses for the left anterior descending coronary artery (LAD) and D2% for 50 Gy and 40.05 Gy groups were 4.86 ± 2.42 Gy, 28.59 ± 11.05 Gy, and 4.27 ± 3.27 Gy, 17.33 ± 10.98 Gy, respectively.

In the ipsilateral lung, the mean lung dose, V5 Gy, and V20 Gy in the 50 Gy and 40.05 Gy groups were 6.61 ± 2.52 Gy, 30.72 ± 9.95%, 13.41 ± 5.91%, and 4.53 ± 1.84 Gy, 23.40 ± 9.73%, 8.86 ± 4.31% Gy, respectively.

### Acute toxicity

All patients completed planned IMPT without interruption. Acute toxicity included the toxicity that happened during and after radiotherapy within three months. The proton therapy-induced acute toxicity is displayed in Table 3. Grade 1–2 hematological toxicities for 50 Gy and 40.05 Gy group were six (42.8%) and 12 patients (33.3%), respectively. Grade 3 hematological toxicities were seen in two patients (14.3%) in 50 Gy group. Grade 1 radiodermatitis developed in five patients (35.7%), and Grade 2 radiodermatitis developed in eight patients (57.1%) in 50 Gy group. One patient presented a grade 3 skin reaction who irradiated 50 Gy in 25 fractions because the patient wore a bust bodice just finished radiotherapy. Grade 1 radiodermatitis developed in 33 patients (91.7%), and Grade 2 radiodermatitis developed in three patients (8.3%) in 40.05 Gy group. There have statistical significant deference in radiodermatitis with two groups.

**Table 3:** Side effect by proton therapy

		50 Gy group	40.05 Gy group	*p*-value
*Leukopenia*				0.441
	Grade 1	2	9	–
	Grade 2	4	3	–
	Grade 3	2	0	–
*Neutropenia*				0.03
	Grade 1	2	2	–
	Grade 2	3	3	–
	Grade 3	2	0	–
*Anemia*				0.201
	Grade 1	0	4	–
	Grade 2	0	0	–
	Grade 3	0	0	–
*Radiodermatitis*				0.000
	Grade 1	5	33	–
	Grade 2	8	3	–
	Grade 3	1	0	–
Radioaction-induced esophagitis	Grade 1	6(85.7%)	3(60%)	0.356
Itching or tingling of the skin		6	7	–
Swelling in the arm		1	2	–
Swelling in the mammary gland		4	2	–
Mammary gland to harden		8	19	–
Breast pain		2	4	–
Radiation pneumonitis				0.230
	Grade 1	0	3	–
	Grade 2	0	1	–
Hypothyroidism		0	1	0.538
Rib fracture		0	2	0.378

Radiation-induced esophagitis only presented in a patient who irradiated the supraclavicular area. In the 50 Gy group, seven patients were irradiated with supraclavicular lymphatic drainage, and six patients (85.71%) presented grade 1 radiation-induced esophagitis. Five patients were irradiated with supraclavicular lymphatic drainage at 40.05 Gy group, and three (60%) developed grade 1 radiation-induced esophagitis. But not found statistical significant deference with two groups.

Six patients reported itching or tingling of the skin in 50 Gy group, while seven reported itching or tingling in 40.05 Gy group.

### Late toxicity

The last follow-up was performed on 31st March 2022, and the total median follow-up time was 38 months (15–68 months). Median follow-up time for 40.05 Gy group was 35.6 months (15–43 months). Median follow-up time for 50 Gy group was 46.8 months (36–68 months).

No patient presented radiation-induced pneumonitis and rib fracture in the 50 Gy group. Three patients presented grade 1 pneumonitis; one patient presented graded 2 pneumonitides and two patients presented rib fracture in 40.05 Gy group. One presented hypothyroidism in 40.05 Gy group, the hypothyroidism apperared after Hashimoto’s thyroiditis diagnosis.

At the last follow-up, 50 patients survived without evidence of disease progression. One stage III patient presented brain metastases without local disease progression; the patient received surgery and X-ray irradiation for brain metastasis. The one- and two-year OS and DFS for 50 Gy group were 100 and 100; 100 and 92.9%, respectively. The one- and two-year OS and DFS for 40.05 Gy group were 100 and 100; 100 and 100%, respectively.

### Cosmetic result

After radiotherapy, no significant changes in breast size or shape were observed in either group. All patients were satisfied with breast shape. Eight (57.1%) cases presented breast hardening in 50 Gy group, while 19 (52.7%) presented breast hardening in 40.05 Gy group.

## Discussion

Although the postoperative adjuvant radiotherapy for breast cancer may reduce local recurrence risk [4, 5], the long-term follow-up data show that radiation may cause a certain increase in heart-related diseases and radiation-induced lung injury. The incidence of radiation-induced heart disease positively correlates with the heart radiation dose. The major coronary event (MCE) increased 7.4% for every 1 Gy increase in mean heart dose, implying that 1 Gy increased mean heart dose caused an increase in the absolute growth rate of MCE from 0.3–0.6% at the age of 80 [6, 7]. A comprehensive analysis from the Early Breast Cancer Trialists Collaborative Group indicated that the lung dose was significantly associated with lung cancer death [14].

Multiple radiation techniques have been used and tried, including optimization beam angle, multi-leaf collimator shielding, intensity-modulated radiotherapy, the prone position, the breath-holding or gating method, and partial breast irradiation to reduce the heart and lung delivery dose. Intensity Modulated Radiation Therapy (IMRT) reduced the high dose area at normal tissue around the target region but still surrounded by large areas of low dose region because the heart and lungs have higher amounts of average dose [15]. Proton radiation therapy is another most appealing approach and enjoys a higher reputation within the breast cancer therapy community due to its inherently physical properties. It can concentrate the delivery dose on the target areas with less exposure to the surrounding normal tissues. Many studies over the years have displayed that proton radiotherapy for breast cancer can significantly reduce the irradiation dose for critical organs [16–18].

Furthermore, photon radiation therapy with tangent field breathing exercises changes the depth of the target edge, and respiratory gating may reduce respiratory movement uncertainty. However, because proton use enfaces the field path in the respiratory movement direction, the path length from the skin to the distal edge does not change significantly with respiratory motion [19]. Thus, the breathing exercises are not a major source of proton treatment uncertainty.

The proton radiotherapy technology has a late start in China, and despite rapid growth, it still has fallen short of the need of patients. Our center was the first in China to introduce proton-heavy ion radiotherapy equipment. Later, it was applied to breast cancer treatment. Meanwhile, some research on proton radiotherapy around Chinese breast cancer patients was initialized in 2017. Our team has accumulated some firsthand data and experiences treating breast cancer through proton radiotherapy. In practice, we found that the arm positioning error could cause a change in the breast tissue thickness, resulting in a change in the posterior border of proton therapy. It results in a lack of target dose or an increase in the heart and lung dose. Therefore, placement repeatability is particularly important in proton radiation therapy. We used a vacuum pad to fix the patient's affected arm, reducing set-up errors (Fig. 1 A). Recently we introduced the C-rad system, using the surface scan technique to increase the placement of repeatability.

The comparative OAR data are demonstrated in Table 4 from our center and other centers. One can notice that the dose in the ipsilateral lung, heart, and left anterior descending branch (LAD) were comparable. It was higher than Cuaron's data, which used rotating gantry multiple-field irradiation technology for breast cancer treatment [20]. Our center used the en face proton field, which could reduce the mean heart dose below 1 Gy. By comparing, we know the multi-angle could further reduce the heart dose. However, whether or not a low dose of less than 1 Gy will affect the heart is unknown; further research is required.

**Table 4:** Comparison of normal tissue dose between our center and other center

	Our center	Sagar A. Patel (2017)		Mirjam E. MAST (2014)				Anna M. Flejimer (2015)			Carmen Ares (2010)			John J. Cuaron
	IMPT	Photon	Proton	IMPT-BH	IMRT-BH	MRT-FB	MPT-FB	Photon with gating	Phton-fb	IMPT	3D-RT	IMRT	IMPT	Proton
Ipsilateral lung*ipsilateral lung*														
V5	30.72 ± 9.95%			7.1 ± 2.7%	21.4 ± 6.6%	21.9 ± 7.1%	7.7 ± 2.7%				27 ± 3%	46 ± 12%	17 ± 7%	4.35(22.5–53.8)
V10	23.38 ± 8.49%							21.5 ± 10.0%	23.3 ± 13.2%	21.1 ± 4.4%				
V20	13.41 ± 5.91%	26.04 ± 4.30%	15.18 ± 3.66%	2.5 ± 1.4%	10.9 ± 4.7%	12.4 ± 5.7%	2.8 ± 1.4%	16.4 ± 8.1%	18.2 ± 10.9%	9.4 ± 1.8%	14 ± 3%	9 ± 1%	7 ± 4%	16.5(6.1–30.3)
Dmean	6.61 ± 2.52 Gy	13.30 ± 1.70 Gy	7.63 ± 1.27 Gy	1.5 ± 0.6 Gy	5.4 ± 1.8 Gy	6.1 ± 2.3 Gy	1.6 ± 0.6 Gy	9.0 ± 3.9 Gy	9.7 ± 5.1 Gy	5.5 ± 1.1 Gy	17 ± 2 Gy	15 ± 2 Gy	7 ± 3 Gy	
*Heart (left breast cancer)*														
D2%	15.40 ± 9.90 Gy							11.0 ± 12.1 Gy	25.4 ± 15.4 Gy	6.0 ± 3.8 Gy				
V5	4.74 ± 2.87%			0.1 ± 0.2%	2.5 ± 2.1%	7.4 ± 4.7%	0.5 ± 0.8%	3.7 ± 3.7%	7.6 ± 4.2%	2.4 ± 1.6%	10 ± 7%	42 ± 9%	2 ± 2%	0.00(0.17–14.40)
V20	1.63 ± 0.85%	1.97 ± 4.40%	0.86 ± 0.68%	0	0.6 ± 0.8%	3.5 ± 3.0%	0.1 ± 0.2%	0.9 ± 1.5%	3.1 ± 2.4%	0.2 ± 0.3%	4 ± 4%	3 ± 2%	0 ± 1%	1.16(0–6.0)
Dmean	0.98 ± 0.63 Gy	2.09 ± 1.48 Gy	0.98 ± 0.66 Gy	0.1 ± 0 Gy	1.5 ± 0.5 Gy	2.7 ± 1.3 Gy	0.2 ± 0.1 Gy	1.6 ± 0.8 Gy	2.7 ± 1.2 Gy	0.4 ± 0.3 Gy	9 ± 4 Gy	12 ± 2 Gy	1 ± 1 Gy	1.0(0.09–3.20)
*LAD (left anterior descending artery)*														
D2%	28.59 ± 11.05 Gy							21.9 ± 16.1 Gy	41.8 ± 8.4 Gy	12.8 ± 3.0 Gy				
Dmean	4.86 ± 2.42 Gy			0.3 ± 0.2 Gy	6.7 ± 5.1 Gy	14.9 ± 9.3 Gy	0.7 ± 0.8 Gy	9.7 ± 9.6 Gy	22.0 ± 11.7 Gy	4.0 ± 0.9 Gy				

*IMPT* intensity-modulated proton therapy; *BH* breath hold; *FB* free breathing; *IMRT* intensity modulated radiation therapy

Additionally, a phase III study (START-2) was carried out by multiple centers in the UK. The clinical outcome and adverse reactions were compared between conventional radiotherapy and hypofractionated radiotherapy for breast cancer. Typically, the median follow-up time is 9.9 years. There were no obvious statistical differences in the 50 Gy and 40 Gy groups based on the ten years' survival rates were identified as 5.5% and 4.3%, respectively. Moreover, there was no significant difference between breast contracture, edema, and capillary expansion. Ischemic heart disease and the incidence of symptomatic pulmonary fibrosis in the two groups were 2.1% and 1.7%, 1.5% and 1.7%, which were almost equivalent. However, the 40 Gy group was better than the 50 Gy group in skin reaction [10]. Proton radiation therapy usually uses 2 Gy of the conventional fraction size; currently, no reports about hypofractionated proton therapy for whole breast irradiation. We retrospectively evaluated 50 women who underwent a lumpectomy and then received proton therapy with the conventional fraction or hypofractionated size in our center. According to the data, the hypofractionated proton radiotherapy presented a better skin reaction than the conventional fractionation dose. There was no adverse reaction of level 3 or above in both groups.

Other academic documents do not report the probability of grade 1 pneumonia after photon radiotherapy of breast cancer. The grade 2 pneumonia after photon therapy is about 2% [15]. Our data demonstrated one patient with grade 2 pneumonia in the hypofractionated group. In addition, two cases presented rib fracture in 40.05 Gy groups. Massachusetts General Hospital (MGH) also reported radiation-induced grade 1, grade 2 pneumonia and rib fracture after proton therapy were 4%, 1% and 7% respectively [21–23]. In clinical practice, the relative biological effectiveness (RBE) usually uses 1.1. However, the distal of the proton beam has the highest linear energy transfer (LET) value. The high LET beam has a high RBE value [24]. In the proton treatment plan for breast cancer, the distal area is at the rib and intercostal muscles, which means at the distal region, the ribs expose higher dose than the treatment plan dose. Thus, we think 2 to 3 beams planning is encouraged to limit the potential biological hotspots and minimize the distal radiobiology uncertainty to ribs (Fig. 1 B–D).

**Fig. 2:**
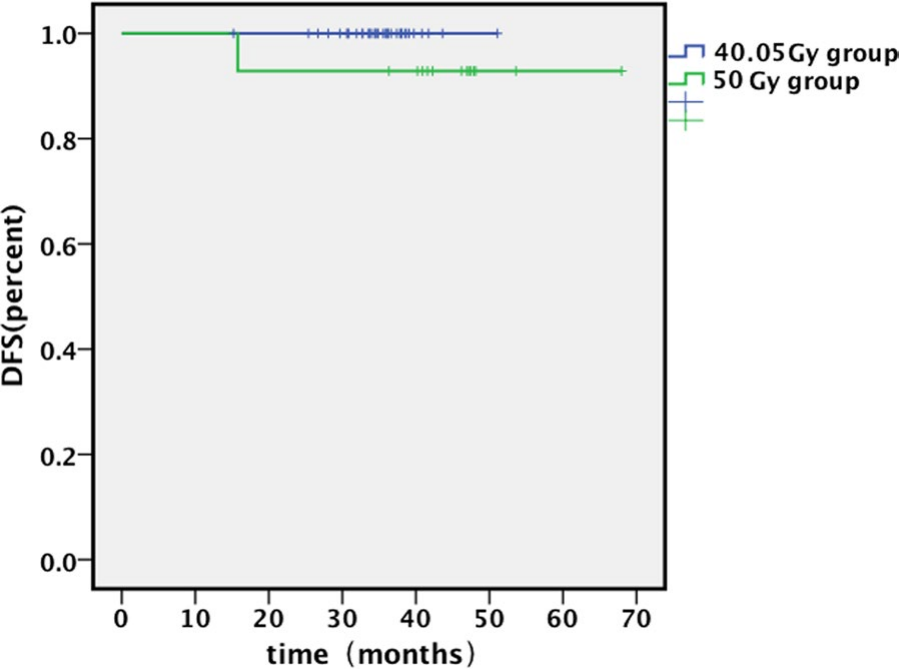
Disease-free survival. Median follow-up time for 40.05 Gy group was 35.6 months (15–43 months). Median follow-up time for 50 Gy group was 46.8 months (36–68 months). The one- and two-year disease-free survival (DFS) rates for the 50 Gy group and 40.05 Gy group were 100 and 92.9: 100 and 100%, respectively

Based on the research results of other centers, we learned that using rotated gantry technology will further reduce the dose distribution of the heart and lung [20]. Because the gantry is fixed at 45 or 90 degrees in our center, we are exploring whether we can achieve a wider irradiation angle by rotating the patient's bed. This statistical data sample quantity for this study is relatively small. Furthermore, there is still no long-term follow-up data for hypofractionated proton therapy for breast cancer. To determine whether proton radiotherapy will eventually reduce late recurrence events cardiopulmonary, secondary malignant tumors, additional investigation on hundreds of patients and decades of close follow-up is still required. Our center is currently conducting a phase II clinical trial of proton therapy for breast cancer, which will provide objective evidence for hypofractionated proton radiotherapy of breast cancer (Fig. 2).

## Conclusion

Proton radiation therapy can significantly reduce the dose of breast cancer patients' hearts, lungs, and other normal tissues. Hypofractionated proton therapy shortens the treatment course with mild radiation-related adverse effects, and has a better effect on addressing the acute adverse reactions than conventional radiotherapy; the late toxicity still needs to be traced and verified by the consecutively continued follow-up research.

## Data Availability

Not applicable.
